# Patterns and Predictors of Premenstrual Symptoms among Females Working in a Psychiatry Hospital

**DOI:** 10.1155/2016/6943852

**Published:** 2016-05-16

**Authors:** Sunita Kumari, Ankur Sachdeva

**Affiliations:** ^1^Department of Psychiatry and Drug De-Addiction, Post Graduate Institute of Medical Education and Research, Dr. Ram Manohar Lohia Hospital, Park Street, New Delhi 110001, India; ^2^Department of Psychiatry, ESIC Medical College and Hospital, NH-3, NIT, Faridabad, Haryana 121001, India

## Abstract

*Introduction*. Premenstrual syndrome presents with vague psychological, somatic, or biological symptoms. It may be seen more commonly in a specific profile of patients. We try to evaluate the patterns and predictors of premenstrual symptoms among females working in a tertiary care psychiatry hospital.* Methodology*. We recruited working females at a tertiary care psychiatry hospital in India, through purposive sampling, and assessed them cross-sectionally. Premenstrual Symptom Checklist was used to assess the frequency and distribution of premenstrual symptoms, which were correlated with various sociodemographic variables to evaluate the predictors for premenstrual symptoms.* Results*. 150 working females were included, belonging to different sociodemographic profile. Somatic symptoms (backache, joint and muscles pain, and fatiguability) were most commonly reported followed by psychological (irritability and losing temper easily) and biological symptoms (increased micturition). Premenstrual symptoms were seen more commonly in women with higher educational status and nursing profession and residing in nuclear families (*p* < 0.05), while age and marital status did not correlate significantly.* Discussion*. Premenstrual symptoms are common and distressing, especially for working females. Somatic symptoms such as backache and joint pains predominate over psychobiological symptoms. Women with higher educational status and professions like nursing belonging to nuclear families are more prone to these symptoms. Attention needs to be given to premenstrual symptoms in such population of working females.

## 1. Introduction

Premenstrual syndrome (PMS) is defined by International Statistical Classification of Diseases and Related Health Problem 10th Revision (WHO, 1992) as occurrence of one distressing premenstrual symptom amongst many symptoms. These include “mild psychological discomfort, feelings of abdominal bloating and weight gain, breast tenderness, swelling of hands and feet, various aches and pains, poor concentration, sleep disturbances, and changes in appetite.” The symptoms should be present during the luteal phase and should cease following the menstrual blood flow [1]. PMS is a broad term which includes a range of physical, emotional, and behavioral symptoms [2].

While PMS impairs the quality of life and social functioning, the presence of only PMS symptoms is mostly not perceived as either distressing or debilitating; hence presence of PMS symptoms is different from a categorical diagnosis of PMS [3]. Premenstrual symptoms are deemed as ranging from mild to moderate in intensity, not particularly debilitating, and not necessarily occurring regularly, while premenstrual syndrome is more severe, involves specific subset of symptoms, occurs relatively regularly, and significantly affects a woman's life [4].

The range of PMS symptoms varies from emotional symptom like anxiety, crying spells, mood swings, and irritability to physical symptoms such as headache, fatigue, abdominal bloating, and breast tenderness [5]. Most of the women suffer only a few of these, although 70–90% of women complain of recurrent PMS symptoms [6]. Due to variations in the universally accepted diagnostic criteria and differences in the interpretation of premenstrual symptoms, there is difficulty in estimating the prevalence of PMS symptoms [7]. Premenstrual symptoms are often underdiagnosed as they are usually not reported by the patient or a clinician often does not ask and has difficulty in diagnosing [8].

PMS symptoms, even though mild to moderate in intensity, might adversely affect and influence daily activities and work productivity [9]. It is more pronounced in cases of working females [10]. Most of the studies done on this subject worldwide focus on women in general population or a specific subset of working females. There are very limited studies that focus on hospital staff and PMS related problems, as this cohort might be more prone to and more affected by specific PMS symptoms. Also, Indian studies on this subject are relatively less and have not discussed PMS in psychiatric hospital population. Most studies have found predominance of somatic symptoms in PMS. We hypothesized that psychological symptoms would be more common than somatic symptoms in psychiatric health care workers, considering the emotionally exhausting work profile. This study was carried out to assess the frequency, pattern, and predictors of PMS symptoms amongst working females in a psychiatry hospital setup in India.

## 2. Materials and Methods

The current study was conducted at a tertiary level psychiatric hospital with postgraduate teaching facility in the state of Ranchi, India. The hospital caters to a large population from the neighbouring states and nearby countries as well. Institutional ethical clearance was taken for the study.

The subjects were selected through purposive sampling technique and assessed cross-sectionally. In present study 150 females were included, who were working in the institute and belonged to different groups consisting of students, doctors, nurses, and attendants. The inclusion criteria included women in reproductive age range of 20–45 years and women who had menstrual periods in the last 6 months, education status of 5th pass or above, and General Health Questionnaire (GHQ-12) score less than 3 and were willing to give informed written consent. The exclusion criteria were females with age more than 45 years, GHQ score more than 3, presence of major gynecological illness in last one year, frequent menstrual irregularities, presence of any acute/severe physical and mental illness, and pregnancy.

A sociodemographic data sheet was used to get primary information regarding the patient. A clinical data sheet was used to inquire about the presence of any physical illness, use of medications, age of menarche, regularity of menstrual period, any consultation with a gynaecologist, taking any oral contraceptives, date of one's last menstrual period, and one's subjective perception of premenstrual tension.

General Health Questionnaire-12 was used to screen for any psychiatric morbidity in normal controls [11, 12]. GHQ-12 is short version of the original General Health Questionnaire containing 60 items. It has a high degree of internal consistency for each of the 12 items with Cronbach's alpha value of 0.37–0.79. Premenstrual Symptom Checklist was used to assess the presence of various feelings and experiences which can possibly occur in relation to premenstrual period. The scale was developed in 1989 in study done at NIMHANS, Bangalore, and standardized in Indian population. It consists of 50 symptoms which are further categorized into three groups: psychological symptoms (18 symptoms), biological symptoms (10 symptoms), and somatic symptoms (22 symptoms) on the basis of their content [13]. The women had to rate the presence and absence of each item during their premenstrual phase. The most common ones of these 10 symptoms were evaluated and correlated with sociodemographic parameters such as age, occupation, marital status, education, and type of family.

The statistical analyses were done with the help of Statistical Package for Social Sciences-17 (SPSS-17). The sociodemographic variables (both continuous and discrete data) were summarized with the help of frequency and percentages. The clinical variables were assessed in similar way. For assessment of different items of various scales, descriptive statistics had been used. For assessing level of significance chi square tests were applied. For correlation of parametric test, Pearson correlation coefficient (*r*) was used.

## 3. Results

The sample consisted of 150 females working in mental health facility. The mean age of menarche of sample population was 13.38 ± 1.48 years.  Table 1 shows the sociodemographic characteristics of the sample population. Among the 150 subjects, 22 (14.2%) were in 15–25 years range, 78 (52.0%) were in 26–35 years range, and 50 (33.3%) were in 36–45 years range. Among the total sample 33 (22.0%) were students, 87 (58.0%) were nursing staff, and 30 (20%) were ward attendants and workers. In terms of educational status, 25 (16.7%) were educated up to primary and middle stages while 125 (83.3%) were educated up to class 10th stage and above. The number of married females was 105 (70%) and 45 (30%) were single. Around 40% had physical illness though it did not warrant any current medical attention.

Table 2 shows the frequency of premenstrual symptoms experienced by the subjects. The premenstrual symptoms were divided and classified into psychological, biological, and somatic ones. A total of 50 symptoms are mentioned on the list. Somatic symptoms were most commonly reported by the study subjects, where 70% females reported backache, 47% reported joint and muscles pain, 40% reported laziness, 38% reported fatigability, 28% reported breast ache and breast engorgement, 26% reported headache, and 24% reported increased body weight. This highlights the importance of somatic presentation of PMS. Psychological symptoms were next in order, 44% reported irritability, 36% reported anger, 26% reported poor efficiency, 24% reported oversensitiveness, and 23% reported symptoms like feeling apprehensive or tense. Biological symptoms were reported least commonly, although more than a quarter of study subjects had biological problems, where 29% reported increased micturition, 24% females reported reduced sexual desire, and 21.5% reported increase in sleep while 23.3% females reported disturbed sleep, 20.7% females reported decreased appetite, and 20% had loose motion.

Most common 10 symptoms that were experienced by the study sample during premenstrual period are highlighted (Table 2). Backache was the most common symptom prevalent in the sample. It was followed in descending order by symptoms of joint and muscles pain, irritability, feeling very lazy and lethargic, fatiguability, losing temper easily, stomachache, increased micturition, breast ache, and poor efficiency.

We tried to correlate the commonest premenstrual symptoms with the sociodemographic parameters of age, educational status, and type of occupation (Table 3). The only significant correlation of age was with irritability. Irritability was found more in females of higher age groups than other two groups (*p* < 0.05). No significant difference was found in other psychological symptoms among these age groups.

The educational status, however, correlated significantly with symptoms of backache, joint and muscles pain, irritability, losing temper easily, and poor efficiency (*p* < 0.05). These symptoms were reported more in the higher education group compared to primary and middle education group. The occupational role had a significant correlation with multiple symptoms. The nursing staff reported significantly more problems relating to backache, joint and muscles pain, irritability, losing temper easily, and increased micturition while the ward workers and attendants had more disturbances due to poor efficiency (*p* < 0.05). Student group did not report any symptom significantly more than other groups.

We found that type of family structure also had an impact on the premenstrual symptoms presentation. Backache was significantly more in females belonging to nuclear families compared to joint families (*p* < 0.05). Similarly, irritability, losing temper easily, and lethargy were also found more in females of nuclear families. Marriage is an event that puts a working female under significant added stress. Poor efficiency and fatiguability were seen significantly more in married working females, compared to unmarried females who were more troubled with symptoms of irritability and losing temper (*p* < 0.05).

## 4. Discussion

The current study is aimed at assessing the frequency, patterns, and the predictors of premenstrual symptoms in females working in a mental health facility. Premenstrual syndrome (PMS) is quite prevalent among women of reproductive age and affects their social functioning, work productivity, healthcare use, and overall quality of life [14, 15]. Conducting this study has a special reference to the context of Indian working women population, especially in health care sector. The study was unique as it was conducted in a mental health facility, where working females are probably more prone to emotional and psychological burnout.

In the present study, somatic symptoms especially joint and muscle pain, backache, and fatigue were more in comparison to psychological and biological symptoms. Such predominance of somatic symptoms has been found by Chandra and Chaturvedi [13] who found changes related to fatiguability, backache, and bloating, that is, more of somatic and vegetative symptoms. More such reports have come from India and Pakistan in general population and in medical setup [16, 17]. A recent Indian study also found similar incidence of backache and somatic symptoms in women in healthcare setting [18]. However, no earlier study has been conducted in psychiatric health care professionals. With this background knowledge, we had presumed that psychological symptoms would be more common than somatic symptoms in psychiatric health care workers, considering the emotionally exhausting work profile. However, the predominance of somatic symptoms shows consistency and predictability in the presentation of PMS.

The present study reported the commonest symptoms of backache (70.7%), joint and muscles pain (47.3%), irritability (44%), laziness (40%), fatigue (38%), loss of temper (36%), and stomach ache (33.3%). Also the symptoms like poor efficiency (26.7%), oversensitiveness (24%), loose motion (20%), decreased sexual desire (24%), disturbed sleep (21.3%), decreased appetite (20.7%), increased sleep (32%), increased micturition (29.3%), headache (26%), increased body weight (24.7%), breast ache (28%), and breast engorgement (28%) were reported frequently. The findings were similar to the study by Samia et al. (2005) and Tatari et al. (2007), who also reported anger, anxiety, backache, depression, fatigue, and general body discomfort to be the commonest symptoms [19, 20].

Reporting of the backache (70.7%) was relatively high in current study, which is in concordance with previous studies [17, 18]. However, Antai et al. (2004) reported only around 14% incidence of backache [21]. The reason for this gross difference in presentation could be the younger cohort (female undergraduate students aged between 16 and 31 years) and nulliparous females, who are less prone to backache as compared to elderly and multiparous females. It can be hypothesized that changes in hormones, neurotransmitters, and prostaglandins along with culturally acceptable sick role attributed to physical symptoms result in such high somatic presentation of PMS [22, 23].

We also tried to find out the predictors of PMS symptoms by correlating the most commonly reported 10 symptoms with the sociodemographic profiles. The intention was to include only significantly reported symptoms (reported by over 25 percent of females) as many of the PMS symptoms had reported rates of less than 10 percent, which would have made the findings obscure and difficult to generalize. We found that premenstrual symptoms were seen more commonly in women with higher educational status and nursing profession and residing in nuclear families (*p* < 0.05), while age and marital status did not correlate significantly with most of the PMS symptoms.

Women with higher age (between 36 and 45 years) had significantly more irritability compared to younger females. The possible explanation could be that irritability in the age group of 36–45 years might be a symptom of nearing menopause [24]. Rest of the PMS symptoms did not differ significantly, although females between 26 and 35 years reported more premenstrual symptoms and changes in interaction, which has been established in previous studies [25, 26]. But in contrast bulk of evidence suggests that PMS is more common among women of younger age [14, 19, 21]. This is attributed to hormonal changes in reproductive age group. In addition to physiologic changes, women's perceptions and expectations about menstruation may show age related changes that alter the type and frequency of cyclical symptoms. Still, there are studies that do not ascribe any importance of age, cognitive attributions, and socioeconomic variables as influencing factors for PMS symptoms [27, 28].

In the present study females with higher education reported significantly more PMS symptoms like loss of temper, irritability, poor efficiency, backache, and joint and muscles pain. Similar to this, Cénac et al. (1987) found PMS frequency was significantly higher among literate women (43%) than illiterates (20%) [29]. These findings are similar to previous studies [30, 31]. Also, it is seen that females with prior knowledge perceive more premenstrual symptoms than females without knowledge. This is also dependent on the nature of reporting which is more easily experienced and expressed by the women with higher education as compared to women with lower education. However, a study did not find any relation of literacy on PMS symptoms as well as management [18]. A limitation of our study is inequitable distribution of subjects who were 10th pass and above (*N* = 125) compared to those who were not (*N* = 25), although this is expected in a hospital setup.

In the present study, occupation of females was an important predictor of PMS symptoms. Almost all of the major 10 symptoms were found significantly more in nursing staff compared to ward attendants and students. However, less commonly reported symptoms such as feeling weepy and crying easily were reported more by students. Others such as headache, agitation, and oversensitiveness along with somatic symptoms of sweating, dizziness, tingling, and numbness were reported more by ward attendants. Since these symptoms were less in frequency, they were not considered and presented in tables. Singh et al. (2004) found depressive symptoms to be higher among student group, teachers, service women, and housewives as compared to doctors and nurses [32]. For symptoms other than depression, the explanation may be the work load as well as attitude towards expression of PMS. It seems that their overall experience due to their work profile is associated with the reporting of PMS. Also, because nurses are more educated than students and attendants, they are more prone to PMS symptoms, which is also reflected in our results. Again, relatively big sample size of nurses might have affected the results.

The role of family also had a big impact on the premenstrual symptoms. Backache, irritability, losing temper easily, and lethargy were significantly more in females belonging to nuclear families compared to joint families (*p* < 0.05). Women who report a greater degree of PMS are found to have increased measures of sad mood and experience more stress [33]. The females in nuclear families have lesser social support and have greater household and work related responsibilities and hence more levels of stress, which may predispose them to PMS. Marriage is an event that puts a working female under significant added stress. Poor efficiency and fatiguability were seen significantly more in married working females, compared to unmarried females who were more troubled with symptoms of irritability and losing temper (*p* < 0.05). Again, the predominance of irritability and mood changes in unmarried younger females may be due to changes in hormone levels during late adolescence and the early 20s.

The study is the first one, to the best of our knowledge, to be conducted among females working in mental health facility, who are often neglected and have their own subset of problems. There is representation in all the categories, which provided the better and appreciable assessment of the data. The wide age range of 16–45 years provides better groupwise assessment of PMS. Also, the sample size of the present study was 150 which compares well with other studies. There are certain limitations to our study. The sample was of convenience and not randomized. Cross-sectional analysis could result in recall bias and more reporting of symptoms in females presently experiencing PMS. Assessment of PMS symptoms for at least two or three menstrual cycles would have helped exclude the episodic occurrence of the symptoms. The severity of PMS and its impact on work could have been studied. Various psychosocial issues, financial factors, lifestyle of females, and existing mental health were not taken into account, which could have influenced the symptomatology.

## 5. Conclusion

The study concluded that PMS is a common and distressing problem in the working women of reproductive age group. Somatic symptoms such as backache and joint pains predominate over psychobiological symptoms, irrespective of the study cohort, although irritability and lethargy are also common. The predominance of somatic symptoms shows consistency and predictability in the presentation of PMS. Women with higher educational status and professions like nursing belonging to nuclear families are more prone to develop these symptoms. Attention needs to be given to varied presentations of premenstrual symptoms in such population of working females. Considering the associated distress and stigma related to menstrual cycle, PMS is an important health issue and needs to be discussed in detail.

## Figures and Tables

**Table 1:** Sociodemographic characteristics of the study sample (*N* = 150).

Serial number	Variables	Subgroups	*n* (%)
1	Age groups (in yrs)	15–25	22 (14.7)
		26–35	78 (52.0)
		36–45	50 (33.3)

2	Occupation	Student	33 (22.0)
		Nursing staff	87 (58.0)
		Ward attendant	30 (20.0)

3	Education	Below 10th standard	25 (16.7)
		10th standard and above	125 (83.3)

4	Family type	Nuclear	110 (73.3)
		Joint	40 (26.7)

5	Religion	Hindu	71 (47.3)
		Muslim	15 (10.0)
		Christian	64 (42.7)

6	Marital status	Married	105 (70.0)
		Unmarried	45 (30.0)

7	Presence of any physical illness		40 (26.7)

8	Using any contraceptive pills		Nil

**Table 2:** Frequency and distribution of premenstrual symptoms (*N* = 150).

Serial number	Psychological symptoms	*n* (%)	Biological symptoms	*n* (%)	Somatic symptoms	*n* (%)
1	Feeling weepy & crying easily	14 (09.3)	Loose motion	31 (20.0)	Restlessness	27 (18.0)
2	Apprehensiveness/restlessness	23 (15.3)	Reduced sexual desire	36 (24.0)	Increased body weight	37 (24.7)
3	Loss of temper^•^	54 (36.0)	Disturbed sleep	32 (21.3)	Headache	39 (26.0)
4	Feeling that people are teasing me	12 (08.0)	Decreased appetite	31 (20.7)	Joint and muscles pain^•^	71 (47.3)
5	Irritability^•^	66 (44.0)	Craving for certain food item	12 (08.0)	Backache^•^	105 (70.0)
6	Sudden mood swing	24 (16.0)	Increased sleep	32 (21.5)	Fatigability^•^	57 (38.0)
7	Making mistake at work	17 (11.3)	Increased sexual desire	12 (08.0)	Laziness^•^	60 (40.0)
8	Forgetfulness	05 (03.3)	Constipation	16 (10.7)	Stomachache^•^	50 (33.3)
9	Poor efficiency^•^	40 (26.7)	Increased micturition^•^	44 (29.3)	Sickness (nausea)	25 (16.7)
10	Poor decision making	07 (04.7)	Decreased micturition	01 (0.07)	Breast ache^•^	42 (28.0)
11	Feeling that people make comment	06 (04.0)			Palpitation	11 (07.3)
12	Crying at small thing and suddenly bursting into tear	15 (10.0)			Sweating a lot	16 (10.0)
13	Feeling ignored	18 (12.0)			Hot and cold flushes	13 (08.7)
14	Oversensitiveness	36 (24.0)			Dizziness	15 (10.0)
15	Thinking about ending life	11 (07.3)			Tremor	06 (04.0)
16	Agitation	12 (08.0)			Tingling and numb sensation	17 (11.3)
17	Clumsiness	01 (0.07)			Feeling bloated up	07 (04.7)
18	Death wishes	01 (0.07)			Swelling in feet	09 (06.0)
19					Breast engorgement	49 (26.0)
20					Lots of white discharge	23 (15.3)
21					Acne	14 (09.3)
22					Burning sensation in private parts	06 (04.0)

• shows most common symptoms.

**Table 3:** Correlation of premenstrual symptoms with sociodemographic variables.

Serial number	Symptom	Age	Educational status	Occupational status				Family type			Marital status		
		*p*	*p*	Student	Nursing staff	Ward attendant	*p*	Nuclear	Joint	*p*	Married	Unmarried	*p*
				*n* (%)	*n* (%)	*n* (%)		*n* (%)	*n* (%)		*n* (%)	*n* (%)	
1	Backache	0.647	0.002^*∗*^	15 (45.4)	77 (88.5)	13 (43.4)	0.001^*∗*^	90 (81.8)	15 (37.5)	0.000^*∗*^	70 (66.7)	35 (77.8)	0.176
2	Joint and muscles pain	0.248	0.023^*∗*^	11 (33.3)	51 (58.6)	09 (30.0)	0.004^*∗*^	51 (46.3)	20 (50.0)	0.695	50 (47.6)	21 (46.7)	0.914
3	Irritability	0.013^*∗*^	0.027^*∗*^	12 (36.3)	42 (48.2)	12 (40.0)	0.020^*∗*^	55 (50.0)	11 (27.5)	0.014^*∗*^	36 (34.3)	30 (66.7)	0.000^*∗*^
4	Feeling very lazy and lethargic	0.550	0.371	12 (36.3)	36 (41.4)	12 (40.0)	0.603	45 (40.9)	15 (37.5)	0.708	47 (44.7)	13 (28.9)	0.071
5	Fatiguability	0.936	0.498	11 (33.3)	35 (40.2)	11 (36.6)	0.362	44 (40.0)	13 (32.5)	0.404	46 (43.8)	11 (24.4)	0.025^*∗*^
6	Losing temper easily	0.208	0.022^*∗*^	09 (27.2)	35 (40.2)	09 (30.0)	0.006^*∗*^	45 (40.9)	9 (22.5)	0.038^*∗*^	30 (28.5)	24 (53.3)	0.003^*∗*^
7	Stomachache	0.728	0.439	11 (33.3)	30 (34.5)	09 (30.0)	0.391	38 (34.5)	12 (30.0)	0.601	32 (30.5)	18 (40.0)	0.257
8	Increased micturition	0.302	0.078	09 (27.2)	28 (32.2)	07 (23.3)	0.048^*∗*^	33 (30.0)	11 (27.5)	0.762	30 (28.6)	14 (31.1)	0.754
9	Breast ache	0.928	0.329	09 (27.2)	26 (29.9)	07 (23.3)	0.661	29 (26.3)	13 (32.5)	0.461	28 (26.7)	14 (31.1)	0.578
10	Poor efficiency	0.154	0.000^*∗*^	06 (18.2)	28 (32.2)	06 (20.0)	0.002^*∗*^	35 (31.8)	5 (12.5)	0.018^*∗*^	34 (32.4)	6 (13.3)	0.015^*∗*^

^*∗*^ *p* < 0.05 is statistically significant.
